# The Aryl Hydrocarbon Receptor as an Immune-Modulator of Atmospheric Particulate Matter-Mediated Autoimmunity

**DOI:** 10.3389/fimmu.2018.02833

**Published:** 2018-12-06

**Authors:** Chelsea A. O'Driscoll, Joshua D. Mezrich

**Affiliations:** ^1^Division of Transplantation, Department of Surgery, School of Medicine and Public Health, University of Wisconsin-Madison, Madison, WI, United States; ^2^Molecular and Environmental Toxicology Center, School of Medicine and Public Health, University of Wisconsin-Madison, Madison, WI, United States

**Keywords:** atmospheric particulate matter, T cells, autoimmune disease, autoimmunity, aryl hydrocarbon receptor, polycyclic aromatic hydrocarbons

## Abstract

This review examines the current literature on the effects of atmospheric particulate matter (PM) on autoimmune disease and proposes a new role for the aryl hydrocarbon receptor (AHR) as a modulator of T cells in PM-mediated autoimmune disease. There is a significant body of literature regarding the strong epidemiologic correlations between PM exposures and worsened autoimmune diseases. Genetic predispositions account for 30% of all autoimmune disease leaving environmental factors as major contributors. Increases in incidence and prevalence of autoimmune disease have occurred concurrently with an increase in air pollution. Currently, atmospheric PM is considered to be the greatest environmental health risk worldwide. Atmospheric PM is a complex heterogeneous mixture composed of diverse adsorbed organic compounds such as polycyclic aromatic hydrocarbons (PAHs) and dioxins, among others. Exposure to atmospheric PM has been shown to aggravate several autoimmune diseases. Despite strong correlations between exposure to atmospheric PM and worsened autoimmune disease, the mechanisms underlying aggravated disease are largely unknown. The AHR is a ligand activated transcription factor that responds to endogenous and exogenous ligands including toxicants present in PM, such as PAHs and dioxins. A few studies have investigated the effects of atmospheric PM on AHR activation and immune function and demonstrated that atmospheric PM can activate the AHR, change cytokine expression, and alter T cell differentiation. Several studies have found that the AHR modulates the balance between regulatory and effector T cell functions and drives T cell differentiation *in vitro* and *in vivo* using murine models of autoimmune disease. However, there are very few studies on the role of AHR in PM-mediated autoimmune disease. The AHR plays a critical role in the balance of effector and regulatory T cells and in autoimmune disease. With increased incidence and prevalence of autoimmune disease occurring concurrently with increases in air pollution, potential mechanisms that drive inflammatory and exacerbated disease need to be elucidated. This review focuses on the AHR as a potential mechanistic target for modulating T cell responses associated with PM-mediated autoimmune disease providing the most up-to-date literature on the role of AHR in autoreactive T cell function and autoimmune disease.

## Background

Currently, there are over 80 recognized autoimmune diseases (1). In the United States alone, autoimmune diseases are among the most prevalent diseases effecting 24.5 million people or approximately 8% of the population (1, 2). Both incidence and prevalence of autoimmune diseases have been increasing worldwide (3–5), however the reasons for these increases remain unknown (4). Autoimmune disease results from failure of cells and tissues to distinguish self from non-self leading to a loss of self-tolerance and autoimmune pathology. Autoreactive T cells play a critical role in development of autoimmune diseases such as type 1 diabetes (T1D), rheumatoid arthritis (RA), multiple sclerosis (MS), and systemic lupus erythematosus (SLE) (6–10). Genetic predispositions account for approximately 30% of autoimmune diseases, leaving environmental factors as a major contributor (11, 12). While genetic predispositions play a role in disease incidence (13), epidemiologic studies strongly support that high levels of air pollution, specifically, particulate matter (PM) in the atmosphere, increase the incidence and severity of autoimmune disease (1, 3).

PM, a component of air pollution, has emerged as the largest environmental risk factor for mortality worldwide (14). While many people equate exposure to inhaled PM with airway disease, its role in other systemic illnesses has also been well documented. Increases in incidence of autoimmune disease have occurred concurrently with increases in global air pollution (3, 4, 14, 15). Exposure to PM has been associated with aggravation of several autoimmune diseases including T1D, MS, RA, and SLE (16–32). Epidemiologic studies strongly suggest that exposure to PM can increase both incidence and severity of autoimmune diseases (33, 34).

Atmospheric PM is a complex mixture of solid particles and liquid droplets formed from a combination of primary sources, such as road transportation (diesel exhaust PM), stationary combustion (mainly domestic coal burning) and industrial processes (35), that emit PM directly into the atmosphere and secondary sources, such as gaseous vegetative emissions, motor vehicle emissions, and wood-smoke emissions (36), that emit gaseous PM precursors into the atmosphere and undergo oxidation reactions to form PM (35, 36). The diverse primary emission sources and secondary chemical reactions that lead to atmospheric PM components result in complex mixtures of PM components that include metals, nitrates, sulfates, and diverse organic compounds like polycyclic aromatic hydrocarbons (PAHs) (37, 38).

PM contains organic compounds such as PAHs and dioxins, among others, which are aryl hydrocarbon receptor (AHR) ligands, adsorbed to its surface (37–40). The AHR is ligand-activated transcription factor that responds to exogenous ligands, as well as endogenous ligands, and upregulates cytochrome P450 (CYP) metabolizing enzymes as well as other gene targets (40). The majority of high affinity AHR ligands are synthetic in nature and include 2,3,7,8-tetrachlorodibenzo-p-dioxin (TCDD), the prototypic AHR ligand, and PAHs, among others (41, 42). The most potent AHR ligands are more metabolically stable, like TCDD and dioxin-like compounds, whereas less potent ligands, like PAHs, are more metabolically labile (41). Early studies of the AHR focused on understanding the underlying mechanisms of TCDD toxicity. It was discovered that TCDD exposure caused severe toxicity and life-threatening manifestations such as progressive liver failure, emphysema, renal failure, and myocardial degeneration, among other pathologies (43). In addition to these manifestations, rodent studies described immune phenotypes of TCDD exposure revealing a role of AHR in the immune system. Following TCDD exposure, rodents suffered profound effects on the developing immune system as well as dose-dependent thymic involution, depletion of other lymphoid organs, and reduced circulating lymphocyte counts (43). The discovery of immune pathologies associated with TCDD exposure led immunologists to focus on the AHR.

The AHR has been studied in many aspects of immunology, but a major focus has been on regulatory and effector T cell differentiation and function. *Ahr* is expressed in most CD4^+^ T cell subsets, with highest expression in T helper (Th)17, type 1 regulatory T cells (Tr1), forkhead box P3 (FOXP3)^+^ regulatory T cells (Treg), followed by Th1 and Th2 (44, 45) and is critical in modulating the balance between Th17 and Treg cells (44, 46). TCDD has been associated with an increase in Treg cells and immunosuppression, whereas other ligands such as 6-formylindolo[3,2-b] carbazole (FICZ), a tryptophan breakdown product, has been associated with enhanced Th17 effector cells and inflammation (44, 46). In the context of autoimmune disease, TCDD has been shown to enhance Treg differentiation and suppress experimental autoimmune encephalomyelitis (EAE), a murine model of autoimmune disease, and FICZ has been shown to enhance Th17 differentiation and worsen EAE (44, 46).

This review summarizes the current research regarding the role of PM on development and/or progression of autoimmune disease. We first provide a brief overview of the role autoreactive T cells play in autoimmune diseases and summarize the evidence that PM impacts T cells and autoimmune disease. Given the numerous and extensive reviews on AHR ligands (40, 47), we only highlight PM-mediated AHR effects *in vitro* and *in vivo*. We then focus on the AHR as the receptor central to the mechanism behind modulating T cell responses in PM-mediated autoimmune disease. We chose to focus on four diseases, T1D, RA, MS, and SLE as strong correlations between PM exposure and worsened disease have been observed and the AHR has been linked to these diseases as well. We examine the data demonstrating the effects of organic constituents adhered to PM, specifically AHR ligands, on T cells and suggest the AHR pathway as a target for modulating PM-mediated autoimmune disease. We propose a novel hypothesis that AHR ligands present in atmospheric PM activate the AHR shifting the T cell balance from regulatory to effector ultimately leading to PM-mediated autoimmune disease.

## The Role of Autoreactive T Cells in Autoimmune Disease

In an attempt to develop a rigorous immune system that can react quickly and decisively to outside insults and internal threats (including bacteria, viruses, and dysfunctional/dysregulated cells), but at the same time to avoid autoimmune insults, multiple non-redundant checkpoints have evolved during the development of immune cells to delete self-reactive lymphocytes and generate self-tolerance (4). Central tolerance eliminates self-reactive T cells during their development by negative selection, however, this process is leaky and some self-reactive lymphocytes escape to the periphery (4, 48). Mechanisms of peripheral tolerance control these autoreactive T cells to avoid damage to cells and tissues through employment of anergy, immunological ignorance, and/or regulation (4). Suppression of autoreactive T cells by Tregs is one critical pathway in the induction of peripheral tolerance (4). Regulatory T cells suppress the actions of effector T cells and control the immune response through cell contact, secretion of inhibitor cytokines, and competition for growth factors (4, 49, 50). Tregs can become overwhelmed by persistent inflammation during an immune response or in some cases are dysfunctional resulting in unregulated effector responses and ultimately autoimmune disease (4, 49, 51–53). The complex development of lymphocytes and random rearrangement of adaptive lymphocyte receptors allows for immense diversity of antigen receptor specificity but comes at the cost of creating self-reactive T lymphocytes that escape to the periphery and evade or overcome peripheral tolerance mechanisms ultimately leading to autoimmune disease (54).

Autoreactive T cells play a role in the pathology of autoimmune disease by overcoming central and peripheral tolerance and rendering Tregs insufficient to dampen inflammatory responses. Genetic predispositions account for less than half of all autoimmune disease leaving environmental factors like PM as a potential contributor to the development of autoreactive T cells. Our studies focus on identifying the active component of PM that exacerbates autoimmune disease and elucidating the mechanism through which it acts. Currently, the focus is on understanding how PAHs present in PM act through the AHR to shift the T cell balance and alter autoimmune disease states.

## Particulate Matter

### Defining PM

PM is a complex mixture of solid particles and liquid droplets that vary in physical and chemical properties as well chemical composition and origin, over time and space (35, 55). PM is defined based on size, specifically aerodynamic equivalent diameter (AED) (56). AED is a measure of behavior of the particle in the air and is a function of particle diameter, density, shape, and surface characteristics (57). The particles are further subdivided into AED fractions based on how the particles are generated and their ability to penetrate and deposit in human airways: PM_10_ (<10 μM), PM_2.5_ (<2.5 μM), PM_0.1_ (<0.1 μM) (56). It is important to appreciate that PM_10_ contains, ultrafine PM_0.1_, fine PM_0.1−2.5_, and coarse PM_2.5−10_ fractions (56). By far, the greatest number of particles fall into the ultrafine size range, PM with an AED of 0.1 μM or less (PM_0.1_) (35). The total number and the total surface area of these particles increases exponentially as the diameter of the particle deceases, and as the diameter of the particle decreases, the total particle mass exponentially decreases (56). PM_0.1_ ultrafine particles are inherently unstable in the atmosphere and coagulate and condensate to form larger particles (35). PM_2.5_ fine particles also grow by coagulation and condensation in the atmosphere (35). PM_10_ coarse particles vary in size and while they contribute little to particle number, they contribute majorly to particle mass (35).

### Composition and Sources of PM

Atmospheric PM is a complex mixture of solid particles and liquid droplets formed from a combination of primary sources that emit PM directly into the atmosphere and secondary sources that emit gaseous PM precursors into the atmosphere and undergo oxidation to form PM (35, 36). These complex mixtures of PM components include metals, nitrates, sulfates and diverse organic compounds like PAHs (37, 38). Anthropogenic primary sources include road transportation (diesel exhaust particulate matter), stationary combustion (mainly domestic coal burning) and industrial processes (35). The nature of the industrial particles depends on the process; however combustion particles are generally dominated by black or elemental carbon and heavy organic materials such as PAHs (58). Secondary sources emit PM precursors, which are gases that lead to PM formation through atmospheric reactions and include gaseous vegetative emissions, motor vehicle emissions, and wood-smoke emissions (36).

### Particulate Matter-Mediated Autoimmune Disease

Increases in global air pollution have occurred concurrently with a dramatic increase in autoimmune incidence (3, 4, 14, 15). Exposure to air pollution, specifically PM, is associated with aggravation of various autoimmune diseases including T1D, RA, MS, and SLE (16–32). Epidemiologic studies strongly suggest that exposure to PM can increase both incidence and severity of autoimmune disease (33, 34).

### PM and Autoreactive T Cells

Exposure to PM has been associated with aggravation of autoimmune diseases including T1D, RA, MS, and SLE, which will be the focus of this review (16–32). Pathology of autoimmune diseases can be mediated by autoreactive T cells and exposure to PM has been shown to alter effector T cell populations in healthy T cells as well as diseased. In human T cells from healthy donors, diesel exhaust particles (DEPs) from low emission diesel engines decreased expression of CD25, a marker for FOXP3^+^ Tregs, on CD4^+^ T cells and induced autophagic-lysosomal blockade *in vitro* which has been associated with pathogenic events of autoimmune disease (59). Using cells from atopy-prone mice, which are highly sensitive hosts, Nakamura et al. (60) showed that nanoparticle-rich DEP reduced cell viability and proliferation in a dose-related manner. Retinoic-acid receptor-related orphan receptor gamma t (RORγt) expression and subsequent IL-17A production/release by the cells was increased in the splenocytes in a dose-dependent manner implicating Th17 cells in PM-mediated immune responses. Additionally, CD4^+^ and CD8^+^ T cells exposed to PM_2.5_ significantly elevated mRNA and protein levels of inflammatory cytokine production in a macrophage-dependent manner (61). Furthermore, in a model of chronically inhaled PM_2.5_ for 24–28 weeks, exposure to PM_2.5_ resulted in increased T cell infiltration and increased activation of effector T cells in the lungs and indicates that PM_2.5_ potentiates a proinflammatory Th1 response (62). In addition, van Voorhis et al. (63) demonstrated that a 3 day intranasal instillation of a standard reference material (SRM)1649b, an ambient urban dust PM sample, significantly upregulated IL-17 mRNA in the lung of C57BL/6 mice. Moreover, in a mixed leukocyte culture, using C57BL/6 splenocytes activated with Balb/c DCs, which creates an immune response, a significant increase in IL-17 protein was measured as well as IL-22 mRNA suggesting an increase in Th17 responses (63). Likewise, Castaneda et al. (64) demonstrated that PM enhances DC activation and primes naïve T cell differentiation toward a Th17-like phenotype *in vitro* and *in vivo*.

### PM and T1D

T1D is an autoimmune disease resulting in targeting of islet cell autoantigens leading to a severe loss of pancreatic β cells (9, 65). T1D patients exhibit defects in peripheral tolerance including effector resistance to Treg suppression (66) and/or impaired Treg function (67). Incidence of T1D has been increasing by 2–5% worldwide (68) especially in children 0–4 years of age (69) and prevalence is approximately 1 in 300 in the U.S. by age 18 (68). Long-term exposure to PM_2.5_ at low levels has been related to increased mortality attributable to T1D (17). Hathout et al. (20) found pre-diagnosis PM_10_ exposure was significantly higher in children diagnosed before 5 years of age compared to healthy controls. Likewise, a study from Chile found that PM_2.5_ levels were associated with the onset of T1D in children (19). Beyerlein et al. (16) analyzed data from a population-based register monitoring incidence of diabetes in children and youths in Germany since 2009 and found that high exposure to the traffic-related air pollutants PM_10_, NO_2_, and possibly PM_2.5_ accelerated the manifestation of T1D, but only in very young children. Additionally, children of mothers exposed to high levels of air pollution while pregnant had a higher risk of later developing T1D (21). Di Ciaula et al. (18) showed T1D incidence rate was significantly and positively correlated with mean yearly PM_10_ in Italy, however the correlation between T1D and PM_10_ air levels was more evident in children 10–14 years and 5–9 years than 0–4 years. Together, these data demonstrate an association between exposure to PM and diagnosis and exacerbation of T1D in children, mainly under the age of 5. On the other hand, a study in Southern California, demonstrated that pre-diagnosis PM_10_ exposure in children was not associated with increased odds of T1D (70). In adult disease, Michalska et al. (71) showed a relationship between the number of new T1D cases and mean annual concentration of PM_10_ in 2016, but not 2015 in Poland. Additionally, Yitshak et al. (72) showed that the 3-month average concentration of PM_10_ was associated with increases of serum glucose, HbA1c (average glucose concentration over 3 months and a marker for diabetic complications), low-density lipoprotein and triglycerides, and decrease of high-density lipoprotein with strongest associations observed among subjects with diabetes. Conversely, Lanzinger et al. (73) found no relationship between PM and T1D and no significant associations between HbA1c and PM_10_. Overall, these data suggest exposure to PM may increase incidence, onset, and accelerate T1D in children and may be associated with worsened diabetes in adults.

### PM and RA

RA is characterized by accumulation of inflammatory cells in the joints, leading to synovitis and severe tissue damage (74, 75). RA is a systemic autoimmune disease effecting approximately 1% of the adult population (76) and overall heritability of RA is estimated to be approximately 60% (77), leaving environmental pollutants, such as PM, as significant factors. Chang et al. (27) detected an increased risk of RA in participants exposed to PM_2.5_ and there are several studies, including the Nurses' Health Study (76), that show an elevated risk of RA in people living less than or equal to 50 meters of major highways (76, 78). However, in the same population of nurses, adult exposures to specific air pollutants were not associated with an increased RA risk (79). Similarly, in the Swedish Epidemiological Investigation of Rheumatoid Arthritis, no evidence of an increased risk of RA with exposure to traffic pollutants, including PM_10_ was measured (80). Nonetheless, there are multiple studies that show an increased incidence of RA in urban areas compared to rural areas (81, 82) and living near air pollution emitters was associated with higher risks of developing RA and of producing RA-specific autoantibodies (82). Additionally, Gan et al. (83) showed that PM exposure was not associated with RA-related autoantibodies and joint signs among individuals without RA, but at an increased risk of developing RA.

Moreover, in children, case control studies indicated an increased relative risk for juvenile idiopathic arthritis (JIA), also known as juvenile rheumatoid arthritis, in American children <5.5 years of age was found with increasing PM_2.5_ exposure, but evidence was less clear for links between exposure to air pollutants and development of RA (84). Furthermore, Zeft et al. (85) demonstrated that increased concentrations of PM_2.5_ in the preceding 14 days of diagnosis were associated with significantly elevated risk of JIA onset in preschool aged children but not older children. Additionally, Zeft et al. (86) showed the most positive associations of short-term PM_2.5_ exposure with systemic JIA were in children younger than 5.5 years. Together these data suggest a strong relationship between exposure to PM and risk of developing RA in both adults and children, however the link between PM exposure and exacerbation of RA is less clear. Overall, these data suggest a potential role of PM exposure in development and/or exacerbation of juvenile and adult onset RA.

### PM and MS

MS is a demyelinating inflammatory disorder of the central nervous system (CNS) mediated by pathogenic T cells against myelin antigens (87). Like other autoimmune diseases, MS has a multifactorial etiology and likely results from an interaction between genetic predispositions, like mutations in the class II major histocompatibility genomic region, as well as environmental factors, like PM_10_ and PM_2.5_. A strong link between risk of MS relapse or hospitalization and concentrations of PM_10_ has been established world-wide (22, 24–26, 88, 89). Additionally, Gregory II et al. (23) found strong associations between total MS prevalence rates as well as individual female and male prevalence rates with mobile sources of PM_2.5_ and PM_10_. Moreover, a significant spatial correlation between the clustering of MS cases and patterns of PM_10_ was found in Tehran, Iran in that significantly higher yearly averages of PM_10_ existed in regions where MS patients lived compared to healthy controls (90). In pediatric MS, poor air quality was related to increased odds of developing MS in the pediatric population (91). For those pediatric patients living less than 20 miles from a recruitment center, the odds for MS increased by 4 as the air quality worsened and similarly, for those living more than 20 miles from the recruitment centers, the odds for MS doubled as air quality worsened (91). To understand how PM may be aggravating MS, Bergamaschi et al. (92) investigated the relationship between PM_10_ levels and inflammatory lesions in the brains of patients with MS using MRI data with gadolinium (Gd), which marks blood brain barrier breakdown and inflammatory lesions, in Italy. They found that PM_10_ levels in the 5, 10, 15, 20, and 25 days before brain MRIs were higher with reference to MRIs of patients with Gd enhanced lesions (Gd+) vs. patients with MRIs having no Gd enhanced lesions (Gd-) and there was a significant association between Gd+ MRI and PM_10_ levels. This suggests that PM exposure may be linked to increased inflammatory lesions and blood brain barrier leakiness and breakdown associated with MS. Furthermore, Klocke et al. (93, 94) exposed pregnant mice to PM and characterized endpoints after birth. Gestational exposure to concentrated ambient fine and ultrafine particles at levels consistent with environmental levels near California freeways altered neuropathology (93). These data suggest that gestational PM exposure alters the developing brain.

Contrarily, using the Nurses' Health Study, there was no relationship found between PM exposure and MS risk for women in the U.S (95). Similarly, Chen et al. (96) found no association between living near a major roadway in MS in patients in Ontario, Canada in a population-based cohort study. In Madrid, Spain no associated was found between PM_10_ or PM_2.5_ and MS emergency room admissions across the period analyzed (97). Despite these studies that did not find an associated between PM and MS risk or exacerbation, a large body of evidence exists suggesting that PM contributes to both onset and exacerbation. Cumulatively, these data demonstrate a potential role of PM exposure in the development and exacerbation of MS, however the specific mechanisms remain unknown.

### PM and SLE

SLE is a caused by an aberrant autoimmune response to unknown autoantigens by both autoreactive T cells and autoantibodies that effect vital organs such as brain and kidney (98). PM exposure has been linked to SLE in both adult and pediatric patients (28–32, 99). In adults, Bernatsky et al. (29) showed that antibodies against double-stranded DNA (anti-dsDNA) and urinary casts, markers of disease related to SLE, were significantly associated with PM_2.5_ levels averaged over 24 or 48 h before clinical visits in Montreal, Canada. Moreover, Bernatsky et al. (30) also demonstrated the odds of having a systemic autoimmune rheumatic disease (SARD), which includes SLE, increased with PM_2.5_ levels (30). Additionally, in two different Canadian provinces, Alberta and Quebec, the odds of being diagnosed with SARDs increased with PM_2.5_ levels (31). Similarly, Fernandes et al. (32) observed a significant increased risk of juvenile-onset SLE disease activity 13 and 16 days after exposure to PM_10_. On days where PM levels exceeded the World Health Organization (WHO) air quality standard (50 mg/m^3^), the risk of juvenile-onset SLE activity was 79.0% higher than it was on days with levels below the standard (32). Together these data suggest a potential role of PM in the development and exacerbation of SLE.

### Summary

Several studies from around the world demonstrate an association between the risk of development and/or exacerbation of autoimmune diseases and exposure to PM, and a few do not (Table 1). While the epidemiological data is strong, the mechanistic understanding of how PM increases the risk of developing autoimmune disease or exacerbates autoimmune disease remains largely unknown. The lack of mechanistic understanding of the components of PM responsible for the epidemiologic correlations as well as the pathways in which the components act, make understanding the contradictory data difficult. Despite the contradictory data, several studies establish clear correlations between exposure to PM and autoimmune diseases. Based on the data presented, one possible mechanism is that PM exposure increases inflammation and exacerbates autoimmune disease, however the specific pathways and mechanisms that are targeted to cause the inflammatory responses are unclear. Identifying components of PM and specific pathways associated with PM-mediated autoimmune disease will allow for targeted therapies to delay onset and ameliorate symptoms caused by PM exposure.

**Table 1:** Summary of the effects of PM and AHR agonists on autoimmunity.

		**Treatment**	**Immunosuppressive**	**Immunostimulatory**	**No effect on autoimmunity**
Pre-clinical	PM	DEP PM		(100)	
		DEP OF	(100)	(100)	
		Ambient PM	(101)	(63, 64)	
		Ambient OF			
	AHR agonists	TCDD	(46, 102, 103)	(104–106)	
		FICZ	(102)	(46, 106, 107)	
		10-Cl-BBQ	(108)		
		Norisoboldine	(109)		
		Tetrandrine	(110)		
		Sinomenine	(111)		
		Laquinimod	(112)		
		ITE	(113)		
		I3C	(107, 114)		
		DIM	(107, 114)		
	AHR knockout		(115–117)		
Clinical	PM			(16, 18–21, 23, 27, 59, 71, 72, 76, 78, 81–86, 90, 92)	(70, 71, 73, 79, 80, 83, 85, 95–97)
	AHR antagonists	GNF351	(118, 119)		

*This table summarizes the effects of PM and other AHR ligands in preclinical and clinical studies based on whether the treatment led to an immunosuppressive or immunostimulatory outcome. Preclinical studies include in vivo animal studies whereas the clinical studies include epidemiology studies as well as studies using human cells or tissue. Most of the studies using AHR agonists led to an immunosuppressive effect whereas PM had both immunosuppressive and immunostimulatory effects. Route of exposure and extent and duration of AHR activation contribute to the effects of AHR ligands on autoimmunity and may explain the differential responses observed in these studies. Several clinical epidemiology data suggest that PM exposure leads to immunostimulatory responses while some suggest it does not have an effect on autoimmunity. Together, these data led to the novel hypothesis that PAHs adhered to PM activate the AHR, shift the T cell balance, and lead to PM-mediated autoimmune disease*.

### The Relationship Between PM and the AHR

Epidemiologic data provides strong associations between PM and autoimmune diseases, however the mechanisms in which PM elicits its negative health impacts are largely unknown. PM contains AHR ligands such as PAHs, dioxins, and polychlorinated biphenyl (PCBs) congeners, among others adsorbed to its surface. Andrysik et al. (120) found that the organic extract of SRM1649a, ambient urban dust PM containing dioxins, PCBs, and PAHs, which are present at the highest concentration of AHR ligands adhered to the sample, as well as its neutral and polar fractions, were potent inducers of AHR-mediated responses. These responses occurred at doses one order of magnitude lower than DNA damage and included induction of AHR-mediated transcription of CYP1A1 and CYP1B1 and AHR-dependent cell proliferation (120). PAHs were major contributors to overall AHR-mediated activity (120). Additionally, extracts of real-world PM_10_ samples obtained from southwest United States and Mexico were rich in PAHs and had significant activity in an ethoxyresorufin-*O*-deethylase (EROD) which measures CYP1A1 induction, and in a luciferase assay, which measures AHR activation (121).

Similarly, when looking at immune cells, den Hartigh et al. (122) examined the effects of PM collected from Fresno, California on activation of human monocytes and found that PM exposure increased CYP1A1 expression, and inhibition of the AHR reduced the CYP1A1 levels and inflammatory responses. Likewise, Jaguin et al. (123) showed that AHR and nuclear factor erythroid 2–related factor 2 (Nrf2) pathways were activated in human macrophages after DEP exposure. Specifically, AHR activation by DEP lowered the capacity of human macrophages to secrete inflammatory cytokines, IL-6 and IL-12p40 (123). van Voorhis et al. (63) demonstrated that a 3 day intranasal instillation of SRM1649b not only increased IL-17mRNA in the lung, but also significantly increased CYP1A1 mRNA *in vivo*. In addition, in a mixed leukocyte culture, where splenocytes from C57BL/6 mice are stimulated with DCs from Balb/c mice to generate an immune response, a significant increase in IL-17 protein levels were observed as well as an increase in CYP1A1 mRNA (63).

When examining the effects of intact PM vs. organic extracts, bioavailability of active components, such as PAHs, has been shown to alter biologic responses. PM samples from complete combustion provided a stronger response in the PAH-CALUX assay, which measures PAH-induced AHR activity, and PM from incomplete combustion provided a weaker response suggesting that PM contains organic components that strongly adsorb PAHs thereby reducing their bioavailability (124). These findings were found to be strongly associated with the amount of elemental carbon present in the PM samples with higher elemental carbon favoring less bioavailability of PAHs in PM (124). Additionally, Libalova et al. (125) demonstrated that exposure to extractable organic matter (EOM) induced significantly lower DNA adduct levels, while expression of AHR-dependent PAH-activating enzymes as well as other AHR target genes, was strongly enhanced compared to benzo[a]pyrene-treated cells. This suggests that the genotoxicity of benzo[a]pyrene is inhibited by other organic compounds bound to PM_2.5_ but induction of AHR-dependent gene expression by benzo[a]pyrene is not inhibited by EOM constituents (125). Vondracek et al. (126) found that although PAHs are major contributors to the AHR-mediated activity of organic compounds associated with particles derived from diesel exhaust, polar compounds, which does include polar PAH derivatives generated through metabolism, present in these mixtures are more active in human cells, as compared with rodent cells. Misaki et al. (127) further demonstrated that polar fractions of DEP contribute significantly to AHR activation *in vitro*. Likewise, Palkova et al. (128) found that PAHs, as well as polar compounds contained within DEP, are important inducers of the AHR-mediated activity and contributed significantly to formation of stable DNA adducts, activation of DNA damage response signaling pathways, and induction of cell death. Together, these data suggest the AHR has the potential to be a modulator of PM-mediated disease.

**Figure 1:**
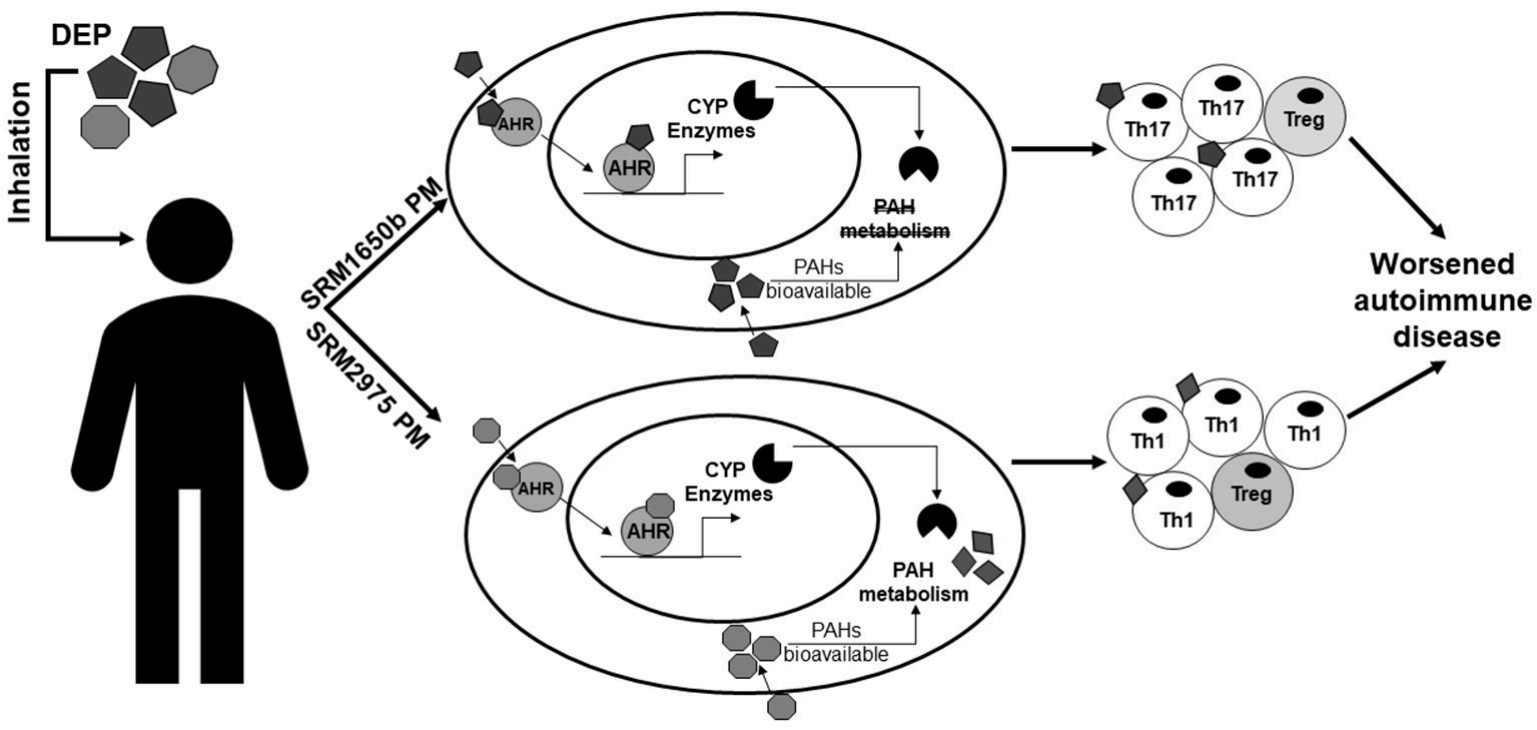
Summary of the effects of DEP on T cells and autoimmune disease. This figure from O'Driscoll et al. (100) summarizes the differential effects of two DEPs, SRM1650b from a 4-cylinder diesel engine, and SRM2975 from a 2-cylinder diesel engine on T cell differentiation and autoimmune disease. SRM1650b enters the T cell, binds AHR, which then translocates to the nucleus and binds DNA, driving transcription of CYP enzymes (**top**). SRM1650b enhances Th17 differentiation in an AHR-dependent manner and worsens autoimmune disease (**top**). Based on the *in vivo* EAE data using the intact PM and chemically-extracted OF, SRM1650b requires the particle to aggravate autoimmune disease because of bioavailability of the PAHs and their ability to activate the AHR. Like SRM1650b, SRM2975 enters the T cell, binds AHR, translocates to the nucleus, binds DNA, and drives transcription of CYP enzymes (**bottom**). However, SRM2975 enhances Th1 differentiation in an AHR-dependent manner but still worsens autoimmune disease (**bottom**). Based on the *in vivo* EAE data demonstrating SRM2975 worsens autoimmune disease in PM and OF forms and the *in vitro* data showing a role of CYP enzymes in T cell differentiation, metabolism of SRM2975 plays a role in its ability to worsen autoimmune disease in that CYP metabolism of PAHs may lead to more potent intermediates that drive immune responses *in vivo*. Additionally, in the presence of PAHs and AHR activation, enhanced effector differentiation by both samples results in increase in Th17 or Th1 cells and a reduction in Treg cells. However, when PAHs are at much lower concentrations as with the low doses, enhanced effector differentiation is lost and Treg differentiation is enhanced. SRM, standard reference materials; DEPs, diesel exhaust particles; AHR, aryl hydrocarbon receptor; CYP, cytochrome P450; PAH, polycyclic aromatic hydrocarbons. This figure or a version of this figure was published in Particle and Fibre and Toxicology and is licensed under the Creative Commons Attribution 4.0 International Public License.

In the context of exposure to PM and its derivatives, O'Driscoll et al. (100) demonstrated that exposure to standard reference material (SRM)1650b PM, which is from a 4-cylinder diesel truck engine, enhanced Th17 differentiation in an AHR-dependent manner and SRM2975, which is from a 2-cylinder diesel forklift engine, enhanced Th1 differentiation in an AHR-dependent manner [Figure 1 from O'Driscoll et al. (100)]. In addition, the chemically-extracted OF of SRM1650b and SRM2975 which contains AHR ligands, such as PAHs, enhanced Th17 differentiation in an AHR-dependent manner (100). Synthetic PAH mixtures which include 15 PAHs present in SRM1650b and SRM2975 enhanced Th17 differentiation, however SRM1650b synthetic PAH mixture required the AHR at high doses and at lower doses enhanced Th17 differentiation only in the absence of CYP enzymes (100). SRM2975 synthetic PAH mixture enhanced Th17 differentiation only in the absence of CYP enzymes suggesting that the inhibition of CYP enzymes prevents the breakdown of the active component allowing for the observed T cell effect [(Figure 1 from O'Driscoll et al. (100)] (100). Similarly, O'Driscoll et al. (101) demonstrated that an ambient urban dust PM sample enhanced Th17 differentiation in an AHR-dependent manner. Likewise, Castaneda et al. (64) showed that PM enhanced DC activation and primed naïve T cells toward a Th17-like phenotype in an AHR-dependent manner *in vitro* and *in vivo*.

### Aryl Hydrocarbon Receptor: an Environmental Sensor

**Figure 2:**
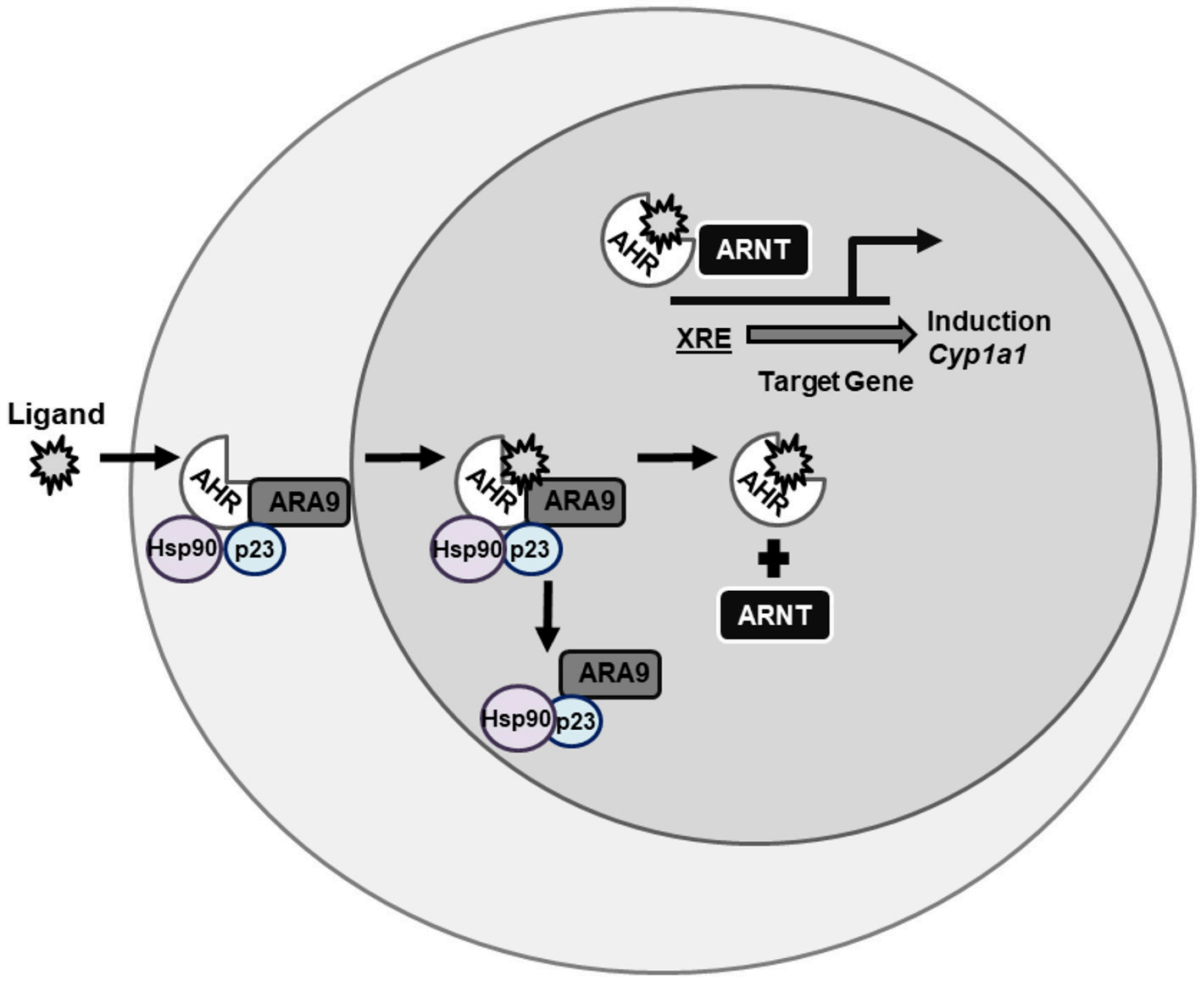
AHR signaling pathway. AHR is a ligand-activated transcription factor the resides in the cytosol being held in conformation by chaperone proteins until bound by ligand. Once bound by ligand, AHR translocates to the nucleus, sheds its chaperone proteins, and binds ARNT. This complex then binds XREs on DNA and induces gene transcription of genes such as CYP1A1. AHR, aryl hydrocarbon receptor; ARNT, aryl hydrocarbon receptor nuclear translocator; XRE, xenobiotic response element; CYP, cytochrome P450.

The AHR is a member of the PER-ARNT-SIM (PAS) superfamily (129, 130) and is a ligand-activated transcription factor that in the absence of ligand is maintained as an inactive complex in the cytosol with two molecules of the chaperone heat shock protein (HSP) 90 (131, 132), as well as aryl hydrocarbon associated protein 9 (ARA9) (also known AIP1 or XAP2) (133, 134) and p23 (135). Together, these chaperones contribute to the cytosolic localization of unliganded AHR, protect it from degradation, and maintain a favorable state for ligand and DNA-binding (136–138). Upon ligand binding, the AHR-complex undergoes a conformational change that reveals its nuclear localization sequence (139). As a result of this conformational change, AHR sheds its cellular chaperones (140, 141), translocate to the nucleus, where it heterodimerizes with another bHLH-PAS protein, aryl hydrocarbon nuclear translocator (ARNT) (also known as HIF1β) (142). The ligand-activated AHR-ARNT complex is capable of binding to specific sequences of DNA (—TNGCGTGT—) known as aryl hydrocarbon response elements (AHREs) [also known as dioxin response elements (DREs) or xenobiotic response elements (XREs)] (143–145) and inducing transcription of target loci such as CYP1A1 (Figure 2) (146, 147). The AHR is a promiscuous receptor that it binds both exogenous ligands, such as TCDD and PAHs, and endogenous ligands, such as FICZ and 2-(1′H-indole-3′-carbonyl)-thiazole-4-carboxylic acid methyl ester (ITE), that are structurally diverse (40, 47, 148).

The AHR is subject to negative regulation. Following ligand-induced activation and nuclear export (141), the AHR is degraded via a 26S proteasome pathway (149–151). Another mechanism in which the AHR is negatively regulated is by the upregulation of the aryl hydrocarbon receptor repressor (AHRR), a bHLH-PAS protein that functions as a transcriptional repressor (152). The AHRR represses AHR transcriptional activity by competing with AHR for heterodimerization with ARNT and from AHRR-ARNT complex binding AHREs (152).

## The Role of AHR in T Cells

AHR is critical in maintaining the balance between Th17 and Treg cells which play a major role in autoimmune disease. TCDD exposure, and subsequent AHR signaling, were shown to play a role in the generation of adaptive CD4^+^CD25^+^ Tregs early in an immune response (153). Additionally, the expression of AHR on T cells was shown to be critical in blocking the generation of peripheral Tregs in the lower gastrointestinal tract after a bone marrow transplant, suggesting the AHR on donor T cells is essential for pathogenesis in acute graft vs. host disease (154). Quintana et al. (46) demonstrated that AHR directly controls FOXP3^+^ Treg generation, by binding AHR binding sites on the *Foxp3* gene. In addition, FICZ, in combination with Th17 promoting cytokines, enhanced Th17 differentiation in an AHR-dependent manner and interfered with the differentiation of Treg cells *in vitro* (46). Moreover, Veiga-Parga et al. (155) showed that a single administration of TCDD reduced severity of stromal keratitis lesions in the cornea by causing apoptosis of FOXP3^−^ CD4^+^ T cells but not FOXP3^+^ CD4^+^ Tregs. TCDD also decreased the proliferation of FOXP3^−^ CD4^+^ T cells resulting in an increase in the ratio of Tregs to T effectors. In addition, *in vitro* studies revealed that TCDD addition to anti-CD3/CD28 stimulated naïve CD4^+^ T cells caused a significant induction of Tregs and inhibition of Th1 and Th17 differentiation (155).

In addition to TCDD, more natural AHR ligands, indole-3-carbinol (I3C), 3,3′-diindolylmethane (DIM), ITE, and kynurenine, among others have been shown to promote Tregs, and FICZ to promote Th17 cells (46, 107, 156–159). Veldhoen et al. (44) demonstrated that AHR is most highly expressed in Th17 cells and AHR ligation by FICZ promotes Th17 differentiation, measured by an increase in percent of IL-17a and IL-22 positive cells, in an AHR-dependent manner. Moreover, Mezrich et al. (156) demonstrated that tryptophan breakdown product kynurenine activates the AHR leading to AHR-dependent Treg generation and has no effect on the generation of Th17 cells (156). Singh et al. (107) showed that treatment of C57BL/6 mice with I3C or DIM attenuated delayed-type hypersensitivity (DTH) response and generation of Th17 cells and promoted Tregs, whereas FICZ exacerbated the DTH response and promoted Th17 cells. Treatment with I3C or DIM decreased the induction of IL-17 but promoted IL-10 and FOXP3 expression in an AHR-dependent manner (107). In addition, Liu et al. (160) demonstrated that FICZ increased Th17 cells and decreased Treg cells, but naphthoflavone decreased Th17 and increased Treg cells.

One question that has emerged as a result of data showing different AHR ligands seem to have opposite effects on T cell differentiation, is whether the ability to elicit different effects is intrinsic to the ligand itself or specific to experimental features, such as route of exposure, AHR affinity, among others? Local administration of FICZ, combined in the emulsion with Complete Freund's adjuvant, worsened EAE (44, 46). However, Duarte et al. (102) demonstrated that systemic administration of FICZ by intraperitoneal injection resulted in partial inhibition of EAE, halfway to what was seen with TCDD. More recently, Erhlich et al. (161) used an acute alloresponse model and demonstrated that when dose and timing of administration of high-affinity AHR ligands was optimized for TCDD-equivalent *Cyp1A1* induction, all ligands tested suppressed the alloresponse and induced Tr1 cells early on and Treg cells later. However, a low dose of FICZ led to transient *Cyp1A1* mRNA expression, no suppression the alloresponse, and enhanced IL-17 production (161). Similar results were observed for low dose TCDD. Together these data demonstrate that route of exposure, dose and duration of AHR activation, and ligand affinity of AHR ligands drives the fate of T cell differentiation and leads to differential T cell effects *in vivo* (Table 1). This suggests that the differential immune responses observed are not intrinsic to each ligand, but rather a result of differential AHR activation.

In addition to FOXP3^+^ Tregs and Th17 cells, AHR has been shown to play a role in Tr1 cells which are FOXP3^−^ regulatory CD4^+^ T cells that produce IL-10 and are generated by IL-27 and have non-redundant roles in the control of inflammation (162). IL-27 also suppresses the development of pathogenic IL-17-producing CD4+ T cells Th17 cells (162). In human PBMCs, AHR promotes the differentiation of Tr1 cells and production of IL-10 through granzyme B (163). Additionally, Mascanfroni et al. demonstrated that at later time points in differentiation AHR promotes hypoxia inducible factor 1-α (HIF1-α) degradation and controls Tr1 cell metabolism.

## The Role of AHR in Autoimmune Disease

In the context of autoimmunity, activation of AHR by exogenous and endogenous ligands modulates T cell differentiation as well as effector and regulatory T cell function, and contributes to antigen presenting cell responses, all of which alter autoimmune diseases. In addition, AHR has been shown to differentially regulate these effector and regulatory T cells through both AHRE-mediated pathways, primarily for Th17, as well as non-AHRE mediated pathways, which have been shown to regulate Tregs (164). Given the complexity of AHR signaling and the differential regulation of T cells, the AHR has been studied as a candidate target for autoimmune disease.

Ishimaru et al. (104) demonstrated that three low dose TCDD exposures in neonatal mice disrupted thymic selection, enhanced production of Th1 cytokines from splenic CD4+ T cells, and increased autoantibodies in the sera of TCDD-exposed mice compared with those in control mice indicating that early exposure to environmental contaminants and consequent AHR signaling in the neonatal thymus alters differentiation and/or development of T cells associated with autoimmunity (104). In addition, Boule et al. (105) found that developmental exposure to TCDD and subsequent activation of AHR via lactation accelerated disease in Gnaq^+/−^ mice, which are mice that are heterozygous for the Gαq protein and have a genetic predisposition to develop an autoimmune disease with symptoms similar to SLE and RA, but are not guaranteed to develop disease. This accelerated disease correlated with increases in effector and regulatory CD4^+^ T-cell populations in females as compared to males (105). Together these data imply a role of early environmental exposure with AHR-mediated autoimmune disease. It has been demonstrated that AHR ligands, including environmental toxicants, bind AHR and alter T cell development and function. In the context of autoimmune disease, AHR has been shown to play a role in T1D, RA, MS, and SLE by altering T cell functions.

### AHR and T1D

One of the most commonly used mouse modes of type 1 diabetes is the non-obese diabetic (NOD) mouse, which develops spontaneous disease similar to humans, and females are most predominately affected (165). Diabetes in NOD mice is characterized by hyperglycemia and insulitis, leukocytic infiltration of the pancreatic islets, and decreases in pancreatic insulin (165, 166). Using NOD mice harboring a transgenic T cell receptor, Bellemore et al. (167) showed that IL-23 plus IL-6 driven Th17 differentiation of CD4^+^ cells results in production of large amounts of IL-22 and these cells induce T1D in young NOD mice upon adoptive transfer. Th17 cells polarized with TGF-β plus IL-6 expressed AHR, IL-10, IL-21, and IL-9, and were able to suppress pathogenic Th17 cells in adoptive transfer experiments suggesting that regulatory T_reg_17 cells induced by TGF-β plus IL-6 express high levels of AHR and are protective while Th17 cells with a very low level of AHR induced by IL-23 plus IL-6 are pathogenic.

Kerkvliet et al. (103) found that chronic treatment of NOD mice with TCDD suppressed the development of autoimmune T1D, reduced pancreatic islet insulitis, and resulted in an expanded population of CD4^+^CD25^+^FOXP3^+^ cells in the pancreatic lymph nodes. However, when TCDD treatment was stopped after 15 weeks, mice exhibited lower number of Tregs and decreased activation of AHR associated with development of diabetes over the next 8 months after treatment was terminated (103). Similarly, Ehrlich et al. (108) discovered that repeated oral dosing with the AHR ligand, 10-Cl-BBQ, suppressed infiltration in islet cells of NOD mice without clinical toxicity in an AHR-dependent manner and this was associated with increased frequency of FOXP3^+^ Tregs in the pancreas and pancreatic lymph nodes. Additionally, depletion of FOXP3^+^ cells did not abrogate immune suppression observed with 10-Cl-BBQ exposure, but reduction of effector T cells was sufficient to suppress disease suggesting 10-Cl-BBQ acts independently of FOXP3^+^ Tregs to suppress the development of pathogenic T cells (108). Additionally, Ehrlich et al. (117) discovered that in the absence of AHR, female NOD mice have a significantly reduced onset of diabetes in comparison to wild-type mice. A similar trend was observed between knockout and wild-type male mice suggesting AHR is important in the onset of T1D in NOD mice (117).

### AHR and RA

RA results in an inflammatory milieu which causes primary human fibroblast-like synoviocytes (FLS) to undergo hyperplasia and ultimately lead to joint destruction. Lahoti et al. (118) demonstrated that co-treatment of FLS with the AHR antagonist, GNF351, repressed IL-1β-induced cytokine and chemokine expression and inhibited the recruitment of AHR to the promoters of *IL-1*β and *IL-6*. In human FLS from patients with RA, the potent AHR antagonist, GNF351, attenuated IL-1β–induced growth factor expression, IL-1β–induced proliferation, protease-dependent invasion, and migration in RA-FLS in an AHR-dependent manner (119). Likewise, the percentage of AHR positive cells in PBMCs as well as AHR and CYP1A1 expression was higher in RA patients compared to healthy controls (168). Additionally, the percentage of AHR^+^CD4^+^CD25^+^ T cell was significantly reduced in RA patients and the percentage of AHR^+^CCR6^+^CD4^+^T cells was significantly increased in patients with RA (168).

Collagen-induced arthritis is a model of RA characterized by infiltration of macrophages and neutrophils into the joint, as well as T cell and B cell responses to type II collagen (169). The model involves immunizing genetically susceptible mice (DBA/1J) with a type II bovine collagen emulsion in complete Freund's adjuvant (CFA) or C57BL/6J mice with type II chicken collagen in CFA and giving a boost of type II bovine or type II chicken collagen in incomplete Freund's adjuvant (IFA) 21 days after the first injection (169). Mice typically develop disease 26 to 35 days after the initial injection (169). A rat model of collagen-induced arthritis involves immunizing with chicken type II collagen and CFA intradermally into the base of the tail on day 0 and a follow-up booster of chicken type II collagen in IFA on day 7 (170).

Isoquinoline alkaloids found in plants have been shown to have AHR activity and induce Tregs alleviating collagen-induced arthritis. Tong et al. (109) demonstrated that norisoboldine, an anti-arthritic isoquinoline alkaloid, functioned as an AHR ligand demonstrated by induction of CYP1A1 expression and activity, promotion of AHR/Hsp90 dissociation and AHR nuclear translocation, induction of AHRE reporter activity, and facilitation of AHR/AHRE binding and promoted intestinal Treg cell differentiation and function in an AHR-dependent manner. Additionally, adoptive transfer of Treg cells from norisoboldine treated mice alleviated arthritis in recipient collagen-induced arthritis mice (109). Similarly, tetrandrine, an alkaloid constituent, alleviated severity of arthritis, reduced serum levels of pro-inflammatory cytokines, and restored the Th17/Treg balance via the AHR, measured by serum levels of IL-17 and IL-10 respectively in collagen-induced arthritis mice (110). Likewise, Tong et al. (111) showed sinomenine, a plant alkaloid, induced the generation of intestinal Treg cells, and facilitated the immunosuppressive function of these Treg cells in collagen-induced arthritis mice. The induction of intestinal Treg cells and the anti-arthritic effect of sinomenine in collagen-induced arthritis mice was diminished by the AHR antagonist resveratrol (111).

Nakahama et al. (115) used a murine collagen-induced arthritis model of RA and showed that AHR deficiency ameliorated collagen-induced arthritis and AHR null mice immunized with collagen showed decreased serum levels of proinflammatory cytokines IL-1β and IL-6. In addition, Th17 cells were decreased in the lymph nodes of AHR null mice whereas Th1 cells in lymph nodes were increased. This loss of AHR specifically in T cells suppressed collagen-induced arthritis development. Further supporting a role for AHR in RA, Talbot et al. (116) demonstrated that cigarette smoke, which contains AHR ligands like PAHs, induces arthritis aggravation and increases the frequency of Th17 cells. Mice null for IL-17 or AHR were protected from cigarette-smoking induced arthritis and exposure to PAHs aggravated arthritis suggesting that AHR ligands in cigarette smoke drive Th17 responses *in vivo* (116).

In RA, the shared epitope (SE), a five amino acid sequence motif encoded by RA-associated *HLA-DRB1* is the most significant genetic risk factor. Fu et al. (106) showed that the SE and the AHR pathway exhibit a synergistic interaction dependent on nuclear factor kappa B (NF-kB) that results in osteoclast differentiation and Th17 polarization after exposure to FICZ or TCDD in bone marrow cells from transgenic mice carrying human SE-coding alleles. *In vivo*, exposure to FICZ or TCDD in transgenic mice carrying human SE-coding alleles resulted in a robust increase in arthritis severity, bone destruction, overabundance of osteoclasts, and infiltration of IL-17-expressing cells in the inflamed joints and draining lymph nodes of arthritic mice (106).

### AHR and MS

Experimental autoimmune encephalomyelitis (EAE) is an inflammatory demyelinating disease of the CNS in rodents that has similar pathologic and clinical symptoms to human MS. In C57BL/6J mice, the disease is induced by myelin oligodendrocyte (MOG)_35−55_ peptide and mediated by CD4^+^ T cells and macrophages (171). Initially, EAE was thought to be mediated by an exaggerated Th1 response, however deficiency in IL-12, and thus IFNγ effector cells, exacerbated EAE (172). In contrast, mice deficient in IL-23, which promotes IL-17 effector cells, failed to develop EAE demonstrating IL-23, not IL-12 as the critical cytokine in autoimmune inflammation (172). Later, Park et al. (173) demonstrated that blocking IL-17 resulted in attenuation and delay of EAE and reversed the progression of active EAE and Harrington et al. (174) showed that IL-23-induced, IL-17 producing CD4^+^ effector T cells have a distinct development program from Th1 or Th2 cells defining them as Th17 cells. Quintana et al. (46) demonstrated that intraperitoneal injection of TCDD shifts the balance toward Treg cells *in vitro* and *in vivo* and suppresses EAE whereas FICZ in the MOG_35−55_ emulsion drives Th17 responses *in vitro* and *in vivo* and worsens severity of EAE (44). However, Duarte et al. (102) demonstrated that intraperitoneal administration of TCDD and FICZ lessened severity of EAE. Using EAE as a model, Kaye et al. (112) showed that laquinimod, an oral drug currently being evaluated for treatment of relapsing and remitting MS, induced genes associated with the AHR pathway such as *Cyp1a1* and *Ahrr* in both naive and EAE mice treated with laquinimod *in vitro* and *Cyp1a1 in vivo*. Laquinimod treatment resulted in an AHR-dependent expansion of Tregs and reduction of effector T cells in EAE (112). O'Driscoll et al. (100) demonstrated that intranasal exposure to diesel PM samples, shown to enhance T cell differentiation through the AHR, worsened severity of EAE, however exposure to its chemically-extracted OF resulted in one diesel OF worsening severity of EAE but the other lost this effect. O'Driscoll et al. (101) demonstrated that mice exposed intranasally to an ambient urban dust PM sample exhibited delayed disease onset and reduced severity of EAE and the delayed disease onset was AHR-dependent *in vivo*. Intranasal treatment to the ambient urban dust PM sample resulted in reduction of pathologic T cells in the CNS on day 10 after EAE induction and in a significant AHR-dependent reduction of IFNγ-producing T cells in an *in vitro* MOG-specific splenocyte assay (101). O'Driscoll et al. (101) identified the AHR pathway as a novel pathway through with PM can reduce Th1 responses in the CNS and although this suppression of Th1 cells may reduce severity of disease it opens the door for opportunistic infection if the immunosuppression in non-reversible. In humans, Rothhammer et al. (175) detected a global decrease of circulating AHR agonists in relapsing-remitting MS patients as compared to controls. However, increased AHR agonistic activity was observed during acute CNS inflammation in clinically isolated syndrome or active MS.

Tr1 cells are regulated by AHR and characterized as FOXP3^−^CD4^+^ T cells that require IL-27, produce IL-10, and have been shown to prevent autoimmune disease. Apetoh et al. (176) demonstrated that AHR activation increased the production of IL-10 and IL-21, which acts as an autocrine growth factor for Tr1 cells, and mice with impaired AHR signaling exhibited decreased production of IL-10 and resistance to IL-27-mediated inhibition of EAE (176). FOXP3^+^ Tregs are also modulated by AHR and play a role in autoimmune disease. Quintana et al. (113) found that AHR signaling participates in FOXP3^+^ Treg differentiation *in vivo* and treatment with the endogenous AHR ligand, ITE, given parenterally or orally induced FOXP3^+^ Tregs that suppressed EAE. Rouse et al. (114) demonstrated that pretreatment of EAE-induced mice with the endogenous AHR ligands, I3C and DIM completely prevented clinical symptoms and cellular infiltration into the CNS and post-treatment of EA- induced mice with I3C or DIM reduced severity of disease. In addition, I3C or DIM promoted the generation of Tregs, while down-regulating the induction of MOG-specific Th17 cells (114).

### AHR and SLE

Rekik et al. (177) demonstrated that transcription of TGF-β1 target genes are impaired in CD3^+^ T cells of active SLE patients and this impaired response to TGF-β1 is associated with an overexpression of IL-22 in SLE patients suggesting that excessive activation of AHR pathway could inhibit the immunosuppressive effects of TGF-β1 leading to exacerbated SLE. Similarly, Dorgham et al. (178) showed a significant expansion of Th17 and Th22 cells in the peripheral blood of active SLE patients, compared to inactive patients and controls. In addition, propranolol, a potential lupus-inducing drug, induced stronger AHR activation in PBMCs of SLE patients than in those of controls and SLE patients also exhibited signs of AHR activation in cutaneous tissues that correlated with lesion expression (178). Moreover, Shinde et al. (179) showed that an enhanced AHR transcriptional signature correlated with disease in patients with SLE. In murine SLE, strength of the AHR signal correlated with disease progression and disease course could be altered by modulating AHR activity (179).

### Summary: AHR Ligands and Autoimmune Disease

Some AHR ligands have been shown to ameliorate autoimmune disease and others to exacerbate disease *in vitro* and *in vivo* (Table 1), but despite these differences it is clear that AHR ligands shift the balance between effector and regulatory T cells determining autoimmune disease outcomes. One question that has been raised is what gives certain ligands the ability to exacerbate disease vs. ameliorate disease? Previously it was thought that AHR regulated Th17 and Treg differentiation *in vitro* and *in vivo* in a ligand-specific manner (44, 46). This created a paradigm that TCDD promotes immunosuppression whereas endogenous ligands like FICZ promote Th17 responses exacerbating immune responses. However, Duarte et al. (102) demonstrated that AHR ligands can upregulate the Th17 program *in vitro* depending on AHR affinity and there are no ligand intrinsic modes of action differentiating one ligand from another. Moreover, *in vivo* the timing and mode of application as well as the differential susceptibility to metabolism by different ligands contributes to the immune response observed (102). More recently, it has been shown that extent and duration of AHR activation contribute to the immune effects observed (161). More specifically, if AHR activation was normalized to TCDD CYP1A1 mRNA induction levels in a model of graft vs. host disease, all ligands tested reduced severity of disease, but if lower levels of TCDD were given, along with other AHR ligands, the exposure increased Th17 cells and started to make disease worse (161). Together, these data suggest that extent and duration of AHR activation determine the immune effect elicited by specific ligands and that ligand-specific responses do not determine the immune responses. Cumulatively, these data demonstrate a clear role of AHR in autoimmune disease and indicate a likely role of AHR ligands present in PM in autoimmune disease.

### Linking PAHS and Autoimmune Disease

PM contains many organic constituents which are AHR ligands that have the potential to contribute to autoimmunity. van Voorhis et al. (63) demonstrated that an individual PAH, benzo[k]fluoranthene, enhanced Th17 differentiation in an AHR-dependent manner. Additionally, PM extracts and cigarette extracts, both of which contain PAHs, enhanced Th17 differentiation as well (63). Although this review focuses on the effects of PM and its constituents on autoimmune disease, it is worth noting that cigarette smoke also contains numerous PAHs at varying levels and there are several epidemiological studies that demonstrate an increased risk of autoimmune diseases such as RA, MS, and SLE in individuals who smoke cigarettes (180–185) and using animal models, Talbot et al. (116) demonstrated that cigarette smoke, which contains AHR ligands like PAHs, aggravates arthritis and increases the frequency of Th17 cells. Furthermore, O'Driscoll et al. (100) demonstrated that synthetic PAH mixtures based on the milieu of 15 PAHs present in standard PM samples enhanced Th17 differentiation via the AHR and/or CYP metabolism. The combination of epidemiological data that associated PM, as well as cigarette smoke, with increased risk and/or exacerbated autoimmune diseases and the *in vitro* studies in mice that demonstrated PM and its chemically-extracted organic fraction (63, 100), cigarette smoke extract (63), individual PAHs (63), and synthetic PAH mixtures (100), enhanced Th17 differentiation suggest that PAHs present in PM are candidate components that may activate the AHR, shift T cell balances, and alter autoimmune disease status.

### Closing the Gap

The incidence and prevalence of autoimmune diseases is continuing to rise and although a clear genetic component exists, environmental factors also contribute to autoimmune diseases. Epidemiologic studies strongly suggest that exposure to PM can increase both incidence and severity of autoimmune disease. Exposure to PM has been associated with aggravation of autoimmune diseases including T1D, MS, RA, and SLE. These autoimmune diseases also have a clear autoreactive T cell component to their pathology. Although a clear role for autoreactive T cells has been established, identifying specific exposures and mechanisms leading to autoimmune disease process has proven particularly difficult.

**Figure 3:**
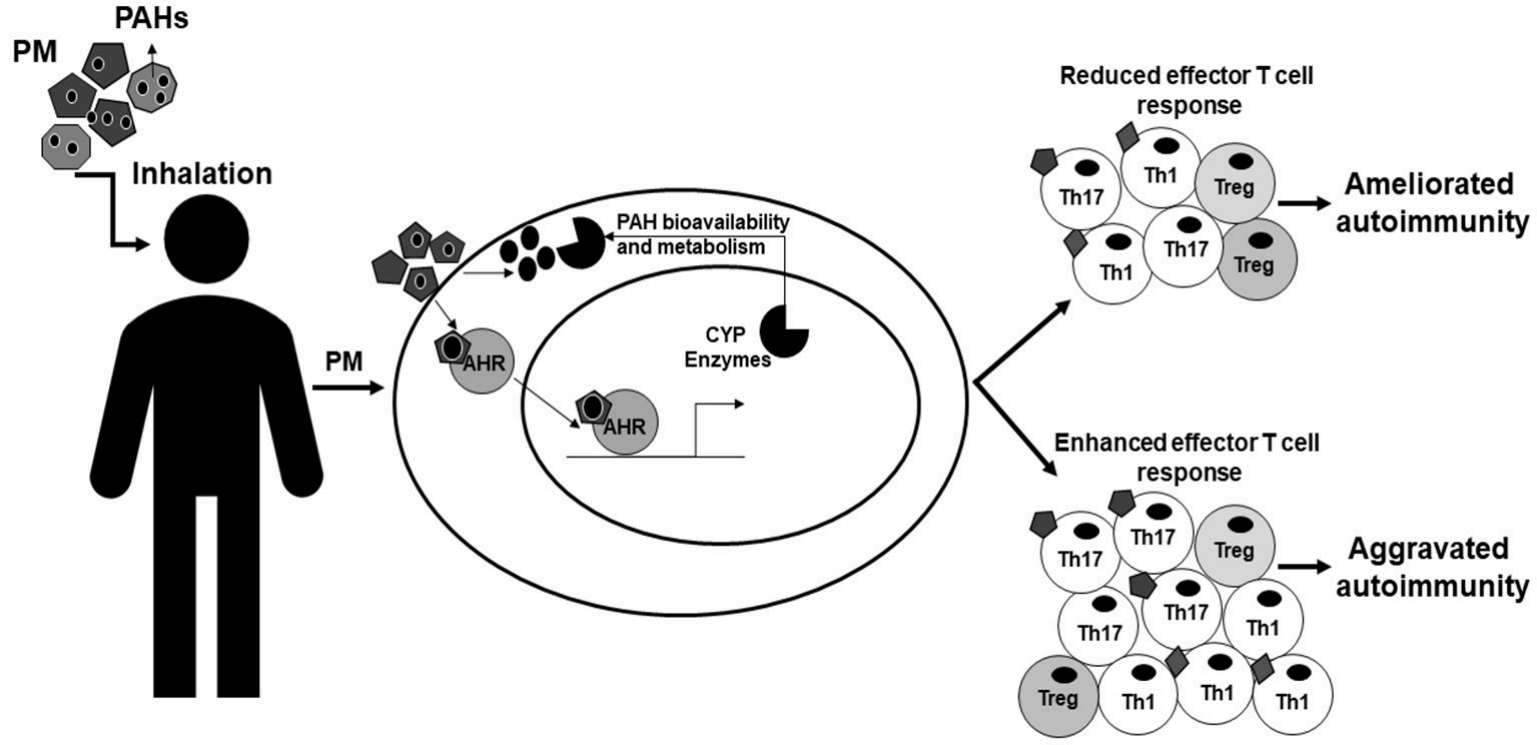
Model of AHR modulation of PM-mediated autoimmunity. This figure is adapted from O'Driscoll et al. (100) and demonstrates how AHR may modulate PM-mediated autoimmunity. PM is inhaled by people and once inhaled is able to be taken-up and has the capacity to activate the AHR in cells within the body. AHR ligands, such as PAHs, adhered to atmospheric PM activate the AHR and cause the AHR to translocate to the nucleus and bind DNA elements such as XRE, inducing genes including CYP enzymes. The extent and duration of activation of AHR ligands shifts the immune balance enhancing effector T cells worsening autoimmunity or suppressing T cell responses and ameliorating autoimmunity. The AHR ligands adhered to the particulate have the potential to become bioavailable through metabolism or other mechanisms and then can be metabolized by CYP enzymes potentially causing other immune related effects or altering the immune responses. AHR, aryl hydrocarbon receptor; PM, particulate matter; PAHs, polycyclic aromatic hydrocarbons; XRE, xenobiotic response element; CYP, cytochrome P450. This figure or a version of this figure was published in Particle and Fibre and Toxicology and is licensed under the Creative Commons Attribution 4.0 International Public License.

PM activates the AHR and induces the production of inflammatory cytokines in immune cells. The AHR can modulate T cells responses by shifting the balance between regulatory and effector responses. Both PM and the AHR have strong associations with autoreactive T cells and autoimmune disease (Table 1). Additionally, PM exposures act through the AHR altering T cell responses *in vitro* and *in vivo* changing autoimmune disease pathology (Table 1). The AHR responds to several environmental toxicants found in PM, such as PAHs, and PM exposures alter T cell balance and autoimmune disease via the AHR. Moreover, the contradictory epidemiologic data could be explained by differences in AHR ligands present in the PM and the extent and duration of activation of the AHR. This leads us to the novel hypothesis that AHR ligands present in PM activate the AHR, shift the T cell balance, and lead to PM-mediated autoimmune disease. We propose a hypothesis in which AHR ligands, such as PAHs, adhered to atmospheric PM activate the AHR and upregulate CYP enzymes [Figure 3 adapted from O'Driscoll et al. (100)]. The extent and duration of AHR activation by AHR ligands adhered to PM shifts the T cell response resulting in an effector T cell response that is suppressed and ameliorates autoimmunity or enhanced and aggravates autoimmunity [Figure 3 adapted from O'Driscoll et al. (100)]. Additionally, AHR ligands may become bioavailable either while adhered to the PM or after getting removed from the PM. Once bioavailable, the ligands have the capacity to get metabolized by CYP enzymes and this metabolism can play a role aggravating autoimmune disease [Figure 3 adapted from O'Driscoll et al. (100)]. The identification of components of PM that activate the AHR and also specific AHR pathway targets that can shift the immune balance from inflammatory to regulatory are crucial for understanding the mechanisms through which the AHR contributes to PM-mediated autoimmune disease. PM contains multiple AHR ligands and thus understanding the specific ligands and pathways involved in autoimmune disease could lend insight into the environmental component of autoimmune pathology. Although the AHR is unlikely to be the whole story, it may be a start to identifying mechanisms to alleviate symptoms of autoimmune disease as well as prevent disease all together. Future studies should investigate (1) the AHR ligands present in PM and how they alter T cell response and (2) the specific AHR pathway components required for responses in order to generate therapies to decrease autoimmune disease and hopefully prevent disease onset secondary to PM exposure.

## Author Contributions

CO and JM wrote the manuscript. All authors read and approved the final manuscript.

### Conflict of Interest Statement

The authors declare that the research was conducted in the absence of any commercial or financial relationships that could be construed as a potential conflict of interest.
